# Comparison of Behaviour in Different Liquids and in Cells of Gold Nanorods and Spherical Nanoparticles Modified by Linear Polyethyleneimine and Bovine Serum Albumin

**DOI:** 10.1155/2014/908175

**Published:** 2014-07-01

**Authors:** Inna A. Pyshnaya, Kristina V. Razum, Julia E. Poletaeva, Dmitrii V. Pyshnyi, Marina A. Zenkova, Elena I. Ryabchikova

**Affiliations:** Institute of Chemical Biology and Fundamental Medicine, Siberian Branch of Russian Academy of Sciences, Lavrentieva Avenue 8, Novosibirsk 630090, Russia

## Abstract

Gold nanorods (GNRs) are considered one of the most promising forms of nanoparticles for nanobiotechnology; however, the problem of their toxicity is currently not resolved. We synthesised GNRs, modified with linear polyethyleneimine (PEI-GNRs), and examined their physicochemical and some biological properties in comparison with GNRs modified with BSA and spherical gold nanoparticles (sGNPs) modified with the same agents. The influence of the buffer, cell culture media, and serum on hydrodynamic diameter and zeta potential of all GNPs was studied. Simultaneously, the size, shape, and formation of a corona were examined by transmission electron microscopy (TEM). PEI-GNRs and GNPs were nontoxic for BHK-21 and HeLa cells (MTT test). Penetration of all GNPs into BHK-21, melanoma B16, and HeLa cells was examined after 30 min, 3 h, and 24 h of incubation using TEM ultrathin sections. PEI-GNRs and PEI-sGNPs demonstrated fast and active penetration into cells by caveolin-dependent and lipid raft-mediated endocytosis and accumulated in endosomes and lysosomes. BSA-modified GNPs showed prolonged flotation and a significant delay in cell penetration. The results show that the charge of initial NPs determines penetration into cells. Thus, the designed PEI-GNRs were nontoxic and stable in cell culture media and could efficiently penetrate cells.

## 1. Introduction 

Gold nanoforms are widely used in biomedical and biotechnological research because of their unique physicochemical properties described in comprehensive reviews [1–5]. The ability of gold nanorods (GNRs) to accumulate in tumours and to convert long-wave light in the transparency window into heat makes this kind of gold nanoparticles (GNPs) particularly attractive for the development of new methods of tumour destruction [6, 7]. GNRs were first synthesised in 1992, and a decade later the synthesis was optimised by the use of cetyltrimethylammonium bromide (CTAB) which defined the rod shape and stabilised the particles [8]. However, the high toxicity of CTAB was a stumbling block to the use of GNRs in biomedicine. To overcome this barrier, the coating of the surface of CTAB-GNRs was modified with various reagents, which reduced the toxicity and provided high stability [9]. Various coatings have been proposed to improve the toxicity of CTAB-GNRs, that is, thiol-modified polyethylene glycol [10], organothiol compounds (3-amino-5-mercapto-1,2,4-triazole and 11-mercaptoundecanoic acid) [11], poly(N-isopropylacrylamide) polymer [12], polystyrene sulfonate sodium salt [13], phosphatidylcholine [14], and Pluronic F-127 [15].

Polyethyleneimine-based polymers represent a group of chemical compounds for the modification of metal nanoparticles (NPs), which provide good biocompatibility to the resulting nanocomposites. Branched polyethyleneimines (PEIs) are mainly used in these studies; however, high molecular weight PEIs (>2 kDa) have high cytotoxicity along with high efficiency of cell transfection [16–18]. In contrast, low molecular weight PEIs exhibit low cytotoxicity and the inability to transfect cells [19]. Similar results were obtained in studies on GNRs modified with high molecular weight PEI (25 kDa). These GNRs showed about 40 times higher efficiency of transfection in comparison with unmodified GNRs/DNA, while the cytotoxicity of the latter was incomparably low [20].

Thus, although various approaches to reduce the toxicity of GNRs have been reported, the problem of obtaining nontoxic GNRs, which are stable in biological fluids and can penetrate into cells, remains valid. We attempted to prepare GNRs that meet these requirements using modification with linear polyethyleneimine (PEI-GNRs). The PEI-GNRs obtained were nontoxic and showed high stability and high dispersion in cell culture media, as well as active uptake by both normal and malignant cells. We also compared the features of PEI-modified rod-shaped and spherical GNPs (sGNPs) with the same NPs modified with bovine serum albumin (BSA), which were used for NP modification [1]. We found that modification of both GNRs and sGNPs with linear PEI resulted in more active uptake by cells in comparison with GNRs and sGNPs modified with BSA, although both BSA and PEI coatings reduced the toxicity of modified GNPs.

## 2. Experiments

### 2.1. Materials

Tetrachloroauric acid (HAuCl_4_·3H_2_O) (Aurat, Russia), sodium citrate solution (Fluka), hexadecyltrimethylammonium bromide (CTAB, >99%) (Fluka), sodium borohydride (NaBH_4_, 99%) (Panreac, Spain), silver nitrate (AgNO_3_, 99.8%) (Fluka), ascorbic acid (99%) (Fluka), and bovine serum albumin (BSA) (66 kDa, Sigma-Aldrich) were used as received. Milli-Q water with conductivity greater than 18 MΩ/cm was used in all experiments. Phosphate buffered saline (PBS) (0.01 M, pH 7.3–7.5, Biolot), 3-(4.5-dimethylthiazol-2-yl)-2,5-diphenyltetrazolium bromide (MTT, Sigma), and dimethyl sulfoxide (DMSO) were used as received. Linear poly(ethyleneimine) (PEI) (87 kDa) in the hydrochloride form was synthesised by A. V. Rogoza (ICBFM SB RAS) from poly(2-ethyl-2-oxozoline), with purity ≥99.5% [21].

### 2.2. Instrumentation

The absorption spectra of colloidal solutions of GNPs in quartz cells (1 cm path length) were registered on a Shimadzu UV-2100 spectrophotometer (Japan). The sizes and zeta potential values for all GNPs (GNPs mean any particles, independently of the shape and coating) were evaluated using a Zetasizer Nano ZS Plus instrument (Malvern Instruments; Malvern, Worcestershire, UK). The zeta potentials of the GNPs were derived from laser Doppler electrophoresis measurements using a DTS1060 cuvette. The Smoluchowski approximation was used to convert the electrophoretic mobility to the zeta potential. Each sample was prepared in triplicate and measured six times at room temperature. The particle size and zeta potential values were averaged over those measurements. Electron microscopic studies of all GNP samples were performed on a JEM 1400 TEM (JEOL, Japan) at an accelerating voltage of 80 kV. For this purpose, the GNP suspensions were adsorbed for 30 s on copper grids covered with formvar film which was stabilised by carbon on a JEE-420 Vacuum Evaporator (Jeol, Japan). The excess liquid was then removed and the grids were examined by TEM. The images were obtained by a side-mounted Veleta digital camera (SIS, Germany). The size of GNPs was measured with a Veleta digital camera using the iTEM program, version 5.2 (Olympus Soft Imaging Solutions, Germany).

### 2.3. Synthesis of Gold Nanoparticles

A suspension of spherical GNPs (sGNPs) was prepared by the citrate method [22]. Sodium citrate solution (5 mL of 38.8 mM) was added to a boiling water solution of HAuCl_4_·3H_2_O (45 mL of 1.1 mM) under intense stirring. After 20 min of stirring, the mixture was cooled down to room temperature, kept for 24 h, and passed through a 0.45 *μ*m filter (Millipore, USA). The resulting suspension of sGNPs was stored at 4°C. The citrate-GNPs had a hydrodynamic diameter of 17.30 ± 2.10 nm; the size measured by TEM was 15 ± 1 nm. The zeta potential was −30.23 ± 2.27 mV and the maximum absorption spectrum was 520 nm.

### 2.4. Synthesis of Gold Nanorods

GNRs were synthesised using a modified protocol of Nikoobakht and El-Sayed [23]. Preparation of the seed solution: an aqueous CTAB solution (5 mL, 0.20 M) was sonicated (20 min, 40°C) in water and mixed with 5.0 mL of 0.25 mM HAuCl_4_·3H_2_O. Ice-cold 0.010 M NaBH_4_ (0.60 mL) was added under stirring to the above solution. After stirring for 1 min, the mixture was left for 30 min at 25°C. This seed solution (CTAB-capped) was further used in the growth stage of nanorod synthesis: HAuCl_4_·3H_2_O (0.5 mL, 0.10 mM) was mixed with CTAB (10 mL, 0.10 M) at 25°C and AgNO_3_ (0.20 mL of 0.60 mM) was then added. To obtain the growth solution, ascorbic acid (100 mL of 0.08 M) was added after gentle mixing. The final step of GNR synthesis was the mixing of the seed solution (12 mL) and the growth solution (10 mL) at 40°C. After 3 h, the resulting mixture was concentrated by double centrifugation at 12000 rpm for 20 min; the procedure also removed the unbound CTAB. The pelleted CTAB-GNRs were dispersed in water, and the suspension was stored at 4°C. The resulting GNRs had sizes of 53.26 ± 4.88 × 20.92 ± 2.77 nm (TEM data), a zeta potential of 43.9 ± 0.98 mV, and the maximum in the absorption spectrum at 760 nm.

### 2.5. Modification of GNRs

The GNRs modified by PEI and BSA were prepared by the versatile layer-by-layer approach [24]. Briefly, CTAB-GNRs (0.5 mL) were added dropwise to a linear PEI aqueous solution (200 *μ*L, 87 kDa, 1 wt%) or to a BSA solution in PBS (10 mM, pH 7.4) under stirring. The mixtures were then stirred vigorously for 6 h. The resultant PEI-GNRs and BSA-GNRs were centrifuged for 15 min (12000 rpm) three times to remove the excess of PEI or BSA. Modified GNRs were stored in aqueous solution (1 mg/mL) at 4°C. The zeta potential values of PEI-GNRs and of BSA-GNRs were 53.03 ± 0.80 mV and −9.15 ± 0.44 mV, respectively. The PEI-GNRs and BSA-GNRs in TEM had sizes of 53.83 ± 7.63 × 19.76 ± 2.76 nm and 56.27 ± 5.33 × 20.19 ± 2.91 nm, respectively.

### 2.6. Modification of sGNPs

The citrate-sGNPs were modified with linear PEI using a modified layer-by-layer technique [20]: the BSA aqueous solution (150 *μ*L, 1 mg/mL) was added to citrate-sGNPs (1.35 mL, 1.16 nM); the mixture was stirred for 1 min and then concentrated by centrifugation (12000 rpm, 10 min). The precipitated sGNPs were redispersed in 50 *μ*L of water and added to a linear PEI solution (200 *μ*L, 87 kDa, 1 wt%) under stirring. After 6 h of incubation, the solution was centrifuged (12000 rpm, 10 min), and the precipitated PEI-sGNPs were redispersed in water (100 *μ*L) and concentrated by double centrifugation at 9000 rpm for 20 min to remove unbound PEI. The resultant PEI-sGNPs had a hydrodynamic diameter of 37.82 ± 2.20 nm and a zeta potential of 34.03 ± 1.00 mV. The diameter measured by TEM was 15.95 ± 2.14 nm. To obtain BSA-modified sGNPs, citrate-sGNPs (0.9 mL of 1.16 nM) were incubated with BSA solution (0.1 mL of 0.01 mM) in PBS (10 mM, pН 7.4) for 1 h at room temperature. The reaction mixture was centrifuged for 15 min (13000 rpm) to remove unbound BSA. The pellet was resuspended in PBS and kept at 4°C. BSA-sGNPs had a zeta potential of −22.40 ± 0.46 mV and a hydrodynamic diameter of 47.10 ± 0.12 nm; the diameter measured by TEM was 13.79 ± 2.76 nm.

### 2.7. Preparation of NP Suspensions in Different Liquids

To wash out possible admixtures, the aqueous suspensions of modified and unmodified GNPs were centrifuged for 10 min at 12000 rpm and the supernatant was removed. To study the behaviour of GNPs in different solutions used in cell cultivation, the pellets of citrate-, BSA-, and PEI-sGNPs and CTAB-, BSA-, and PEI-GNRs were diluted (1 : 9 to a concentration of 0.04 mg/L) with (1) Dulbecco's Modified Eagle Medium (DMEM, Gibco BRL, Germany) supplemented with 10% FBS (Gibco BRL, Germany), (2) conditioned DMEM (collected from Madin-Darby canine kidney epithelial cells (MDCK) after 48 h of cultivation at 37°C with 5% CО_2_), (3) 10% aqueous solution of FBS, and (4) PBS. The resulting suspensions were sonicated for 5 min. After 30–40 min (the time required to prepare GNPs for cell culture experiments), all samples were subjected to DLS examination and then incubated at 37°C for 24 h.

### 2.8. Cell Cultures

Cultures of B16 melanoma cells, baby hamster kidney cells (BHK-21), and cervical carcinoma cells (HeLa) were purchased from the cell collection of the Institute of Cytology of Russian Academy of Science (St. Petersburg, Russia) and propagated at 37°C in DMEM supplemented with 10% heat-inactivated FBS, penicillin (100 U/mL), streptomycin (100 *μ*g/mL), and amphotericin (0.25 *μ*g/mL) in a humidified atmosphere containing 5% CO_2_. The same conditions and media were used in all experiments with all cell lines used in this study.

### 2.9. Cytotoxicity Assay

The cytotoxicity of GNPs was evaluated by the MTT test [25], which revealed the amount of viable cells. B16, BHK-21, and HeLa cell lines were seeded into 96-well plates (3 × 10^3^cells per well) and incubated for 24 h in 100 *μ*L of DMEM with 10% FBS. The medium in wells was then replaced with 100 *μ*L DMEM containing 10% FBS and all studied GNPs at concentrations from 2 to 0.0002 mg/L. The cells were incubated with GNPs for 24 or 48 h and then MTT was added to the wells at a final concentration of 0.5 mg/mL; incubation with MTT was then continued for another 3 h. After the media were removed, DMSO (100 *μ*L) was added to each well. The absorbance of samples was measured at 570 and 620 nm using a Multiscan RC (Labsystems, Finland). All experimental points were run in four parallels. Results are represented as the percentage of living cells compared to control cells (untreated with GNPs). The number of control cells was set at 100%.

### 2.10. In Vitro Studies

B16, BHK-21, and HeLa cells were seeded in 6-well plates (1 × 10^5^ cells per well) and incubated for 24 h in 1 mL of DMEM containing 10% FBS. The medium was then removed, and 1 mL of the PEI-sGNP, BSA-GNR, or PEI-GNR suspension was added to B16 and BHK-21 cells. HeLa cells were treated with 1 mL of citrate- or BSA-sGNP suspension. Before application to cells, the suspensions of all GNPs were centrifuged (10 min, 12000 rpm), and the pellets were dispersed in DMEM containing 10% FBS to a concentration of 0.05 mg/L. Cell lines treated with different GNPs were incubated for 30 min, 3 h, and 24 h. Untreated cells served as the control. After incubation, cells were collected from wells with trypsin and pelleted by centrifugation at 6000 rpm for 5 min, before being fixed with 4% paraformaldehyde at 4°C. The samples were routinely dehydrated in ethanol and acetone and embedded into an epon-araldite mixture. Ultrathin sections were prepared using a diamond knife (Diatome, Switzerland) on an ultramicrotome EM UC7 (Leica, Germany), routinely contrasted with uranyl acetate and lead citrate, and examined using a JEM 1400 TEM.

## 3. Results and Discussion

### 3.1. Physicochemical Characteristics of GNPs

It is well known that NPs actively interact with components of physiological fluids, mainly proteins contained therein [5, 26–28]. Many studies of NPs have been performed using cell cultures, which are propagated in media containing serum, which in turn contains a variety of proteins and other biomolecules. Whereas branched PEIs [29] and BSA [7, 14] are widely used in biotechnological studies, no data on the application of linear PEI have been published. We first used linear PEI for the modification of toxic CTAB-GNRs and examined the properties of the resulting NPs.

**Figure 1:**
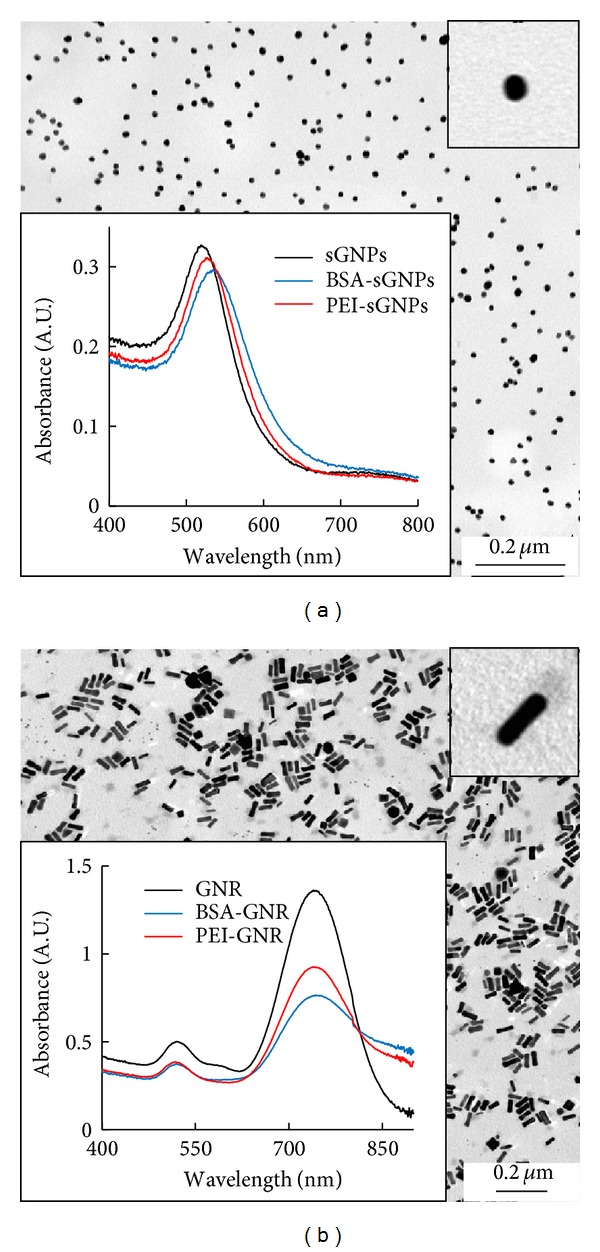
Transmission electron microscopic images and absorption spectra of citrate-sGNP (a) and of CTAB-GNR (b) suspensions in water.

Firstly, we examined the initial suspensions of citrate-sGNPs (Figure 1(a)) and CTAB-GNRs (Figure 1(b)) which appeared homogeneous and highly dispersed in TEM. Then, we studied how the PEI coating could alter surface plasmon resonance parameters, which are essential physical characteristics of GNPs. The absorption spectra of GNPs were found to be identical, before and after modification, with PEI or BSA (Figure 1), indicating the colloidal stability of modified GNPs suspensions.

In order to examine the behaviour of PEI-coated GNRs and sGNPs in the liquids used for cell propagation, we incubated them at 37°C for 24 h in (1) DMEM with 10% of FBS (DMEM), (2) conditioned DMEM containing 10% of FBS (conditioned DMEM), (3) a 10% aqueous solution of FBS (FBS solution), and (4) phosphate buffered saline (PBS). For comparison, we incubated BSA-modified sGNPs and GNRs under the same conditions. The initial aqueous suspensions of PEI- and BSA-GNPs, citrate-sGNPs, and CTAB-GNRs were not incubated at 37°C.

**Figure 2:**
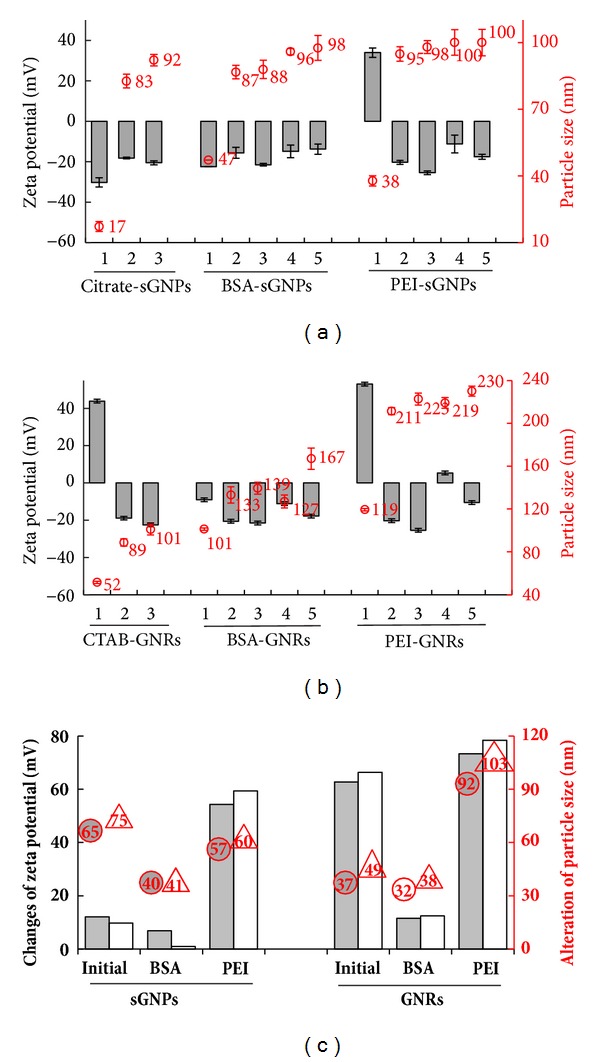
Characteristics of gold NPs obtained by dynamic light scattering. (a), (b) Zeta potential values (gray bars, left axes) and particle size (circles, right axes) of GNPs in different liquids: (1) initial GNPs in water, (2) DMEM, (3) conditioned DMEM, (4) 10% FBS, and (5) PBS. (c) Absolute value of changes in the zeta potential (gray bars, left axes) and particle size (circles, right axis) values of GNPs in DMEM (gray bars) and conditioned DMEM (white bars) in comparison with initial GNPs (citrate-sGNPs and CTAB-GNRs) in water. The left block corresponds to DMEM and the right block corresponds to conditioned DMEM. All values are averaged over three experiments.

We applied the fixed angle dynamic light scattering (DLS) method for the characterisation of modified GNPs in different media. It should be noted that we used the DLS method for both spherical and rod-shaped GNPs, while most publications have reported the application of DLS only for spherical NPs. Only Liu and coauthors have recently shown that DLS can be successfully applied to GNR characterisation [30]. We found that the PEI and BSA modification of GNPs and GNRs increase hydrodynamic sizes and in some cases result in changes in zeta potential (Figure 2). Characteristics of GNP suspensions in culture mediums, FBS solution, and PBS after 30–40 min of incubation did not differ from those after 24 h of incubation.

According to the DLS data, the size of spherical citrate-GNPs increased by additional 65 nm and 75 nm after incubation in DMEM and conditioned DMEM, respectively. Modification with PEI did not alter this trend (Figure 2(c)). Similarly, the hydrodynamic size of BSA-modified citrate-sGNPs increased by very similar values (over 30–40 nm) in DMEM and conditioned DMEM. It was found that initial citrate-sGNPs had negatively charged layers in all liquids (Figure 2(a)). Their modification with PEI yielded positively charged particles with a high zeta potential of 34.03 ± 1.32 mV, following observation in distilled water. Incubation of PEI-sGNPs at 37°C for 24 h led to a change of the initially positive zeta potential to a negative one in culture media, FBS solution, and PBS. Zeta potentials of sGNPs modified with PEI and BSA were close in value and charge when placed in culture DMEM and remained negative in conditioned DMEM (Figure 2(a)).

The hydrodynamic size of rod-shaped CTAB-GNPs increased by 37 nm and 49 nm in DMEM and conditioned DMEM, respectively (Figure 2(c)). CTAB-GNRs with the initially positive zeta potential (43.86 ± 1.89 in distilled water) acquired a negative charge when incubated in culture media. PEI coating retained a high zeta potential of 53.09 ± 1.70 mV in distilled water, which was reduced to 5.35 ± 1.54 mV in FBS solution (Figure 2(b)). Placing of PEI-coated nanorods in culture media changed the charge to negative. It should be noted that relatively large and negatively charged PEI-GNRs in the serum-containing media (fresh and conditioned) displayed a significant increase in size by 90–100 nm in comparison with initial CTAB-GNRs and BSA-GNRs (Figure 2). The zeta potential of BSA-GNRs increased in absolute values as compared with water-diluted BSA-GNRs, which remained negative. Zeta potential of BSA- and PEI-coated gold nanorods was negative and similar in value when GNRs were placed in DMEM and insignificantly changed upon dilution in conditioned DMEM.

Thus, our results indicate that PEI and BSA coatings of spherical and rod-shaped GNPs enhance their hydrodynamic size. We found that hydrodynamic sizes of PEI- and BSA-gold nanorods increased substantially compared with CTAB-GNRs in the same liquids, while the hydrodynamic sizes of various spherical GNPs were similar to the initial sGNPs. It should be noted that the placement of both sGNPs and GNRs in culture media necessarily led to an increase in hydrodynamic size, as shown by DLS. These results may be explained by the adsorption of proteins from the FBS-containing media on the NPs surface [28, 31] or by the aggregation of NPs in these liquids. The values of zeta potential of GNRs ranged from −15.57 ± 1.72 mV to −25.01 ± 0.92 mV with or without PEI and BSA coating when these GNRs were diluted in DMEM and conditioned DMEM; in this case, functionalization of GNRs did not alter the behaviour of PEI- and BSA-GNRs in culture media in comparison with initial CTAB-GNRs.

### 3.2. TEM Analysis of GNPs Behaviour

**Figure 3:**
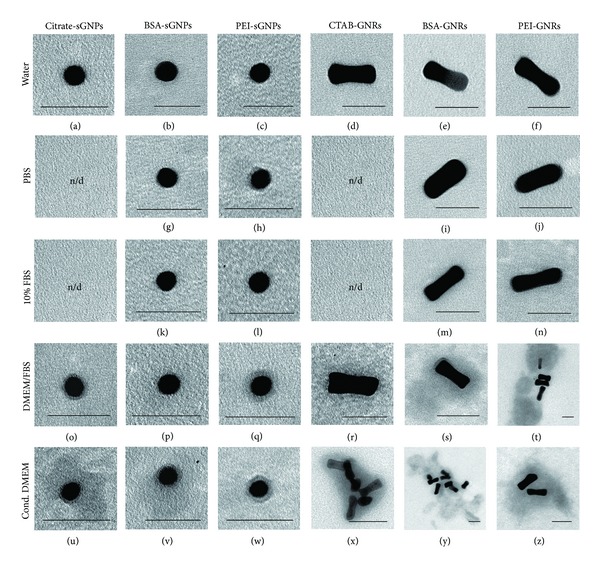
Representative images of GNPs in different liquids (TEM of adsorbed suspensions). The type of GNPs is indicated on the horizontal line and the type of liquid is indicated on the vertical line. Scale bars correspond to 50 nm.

Electron microscopic analysis provided information about the size and shape and aggregation of NPs and shells on their surface, which is useful for the development of preparations based on NPs. We found that citrate-stabilised spherical GNPs aggregated with the formation of agglomerates in DMEM, and small and large aggregates were formed in conditioned DMEM and PBS, respectively. Growth of the hydrodynamic diameter of these particles is explained by the formation of some covering layers on the surface of citrate-sGNPs in DMEM (Figure 3(o)) and conditioned DMEM (Figure 3(u)), as detected by TEM.

It was interesting to compare the behaviour of PEI-modified GNPs with other GNPs in different liquids providing different environments. PEI-modified spherical GNPs were found to be highly dispersed and adsorbed on a grid as individual particles when diluted in water, fresh and conditioned DMEM, and FBS solution (Table 1). According to DLS data, increases in the hydrodynamic size of PEI-sGNPs when water was replaced by protein rich medium may be explained by NPs being coated with some material containing FBS or by interactions between coexisting components and particles in the suspended state. The PEI-sGNPs had no visible coating in distilled water; therefore, we excluded the influence of this parameter on size increase. Using TEM, PEI-sGNPs were found to acquire a corona in PBS (Figure 3(h)), DMEM (Figure 3(q)), and conditioned DMEM (Figure 3(w)). A corona appeared as a distinct layer of material with a thickness of less than 7 nm and an average electron density around NPs similar to that previously described [28, 32]. Also, some of the PEI-sGNPs in conditioned DMEM were coupled with clumps of medium material by segments of a particle. Taking these into account, we can conclude that increases in hydrodynamic size may be related to some agglomeration of PEI-sGNPs (in 10% FBS and PBS) and NP coating in culture media (in DMEM and conditioned DMEM).

BSA-modified spherical GNPs remained naked in most liquids: in water (Figure 3(b)), PBS (Figure 3(g)), 10% FBS (Figure 3(k)), and DMEM (Figure 3(p)). The term “naked” indicates that no substance was seen around the particles by TEM. BSA-modified NPs were highly dispersive in all liquids, except PBS, and were adsorbed on the grid as individual particles (Table 1). Coronas were observed around BSA-sGNPs only in conditioned DMEM, and many particles were surrounded by a layer of material which was significantly thicker (20–70 nm) and had an irregular shape (Figure 3(v)). Our results suggest that the increase in hydrodynamic size of BSA-modified sGNPs may be related to the linkage of few particles in solution, or in the case of conditioned DMEM, to the formation of a layer around the particles.

The suspension of CTAB-GNRs was highly dispersed, not only in distilled water but also in culture media. However, biological applications of GNRs are impossible due to the strong cytotoxicity of the covering surfactant. The hydrodynamic size of CTAB-GNRs (Figure 2(b)) corresponds to the formation of a corona in DMEM (Figure 3(r)) and 20–40 nm layers around particles in conditioned DMEM, as well as some aggregation (Figure 3(x)).

In contrast, PEI-coated gold nanorods agglomerated in water, 10% FBS, and DMEM, while PEI-GNRs were found as individual particles in conditioned medium (Table 1). Some PEI-GNRs were associated with clumps of the material with an average electron density in DMEM (Figure 3(t)) and conditioned DMEM (Figure 3(z)) and were naked in other liquids. These data correspond to the increase in PEI-GNR hydrodynamic diameter caused by agglomeration or coating formation. It should be noted that the morphology and thickness of PEI-GNRs enveloping layers differ from those of their spherical counterparts; the corona around PEI-coated spherical GNPs appeared to be more uniform. The presence of a corona around PEI-GNRs may be dependent on the size, shape, and aspect ratio, which determines the surface area [27, 28].

BSA-modified gold nanorods showed an increase in hydrodynamic size in DMEM and conditioned DMEM due to the formation of coronas and a thick layer (20–50 nm), while the size of BSA-NRs diluted in water, PBS, and FBS solution was dependent on agglomeration processes. Special attention should be paid to the high adhesion of BSA-GNRs to material with an average electron density of those present in DMEM (Figure 3(s)) and conditioned DMEM (Figure 3(y)).

Thus, detailed TEM analysis of GNP suspension enabled us to augment data of DLS and explain some aspects of GNP behaviour in compositionally different liquids. As a result, the dilution of spherical GNPs and GNRs modified with PEI or BSA in conditioned DMEM has been found to prevent agglomeration, which agrees with previously reported data [33, 34]. It should be noted that published electron microscopic descriptions of corona varieties are poor, and “corona” refers to a detectable layer of any thickness in physicochemical studies [35, 36]. However, a clear difference was seen between the corona in Figure 3(q) and the corona in Figure 3(u). We believe that it is possible to distinguish, in addition to the corona, another variant of the material, which is visible on the surface of the NPs at the electron microscopic level; we call this a “cloud.” Thus, clouds are irregular layers of material around individual NPs or their agglomerates with a thickness of more than 10 nm and an average electron density. The binding of a substance in liquid with surface of a particle seems to be a complicated event, which depends on many different factors, described as the “behaviour” of NPs.

### 3.3. Cytotoxicity Assay

**Figure 4:**
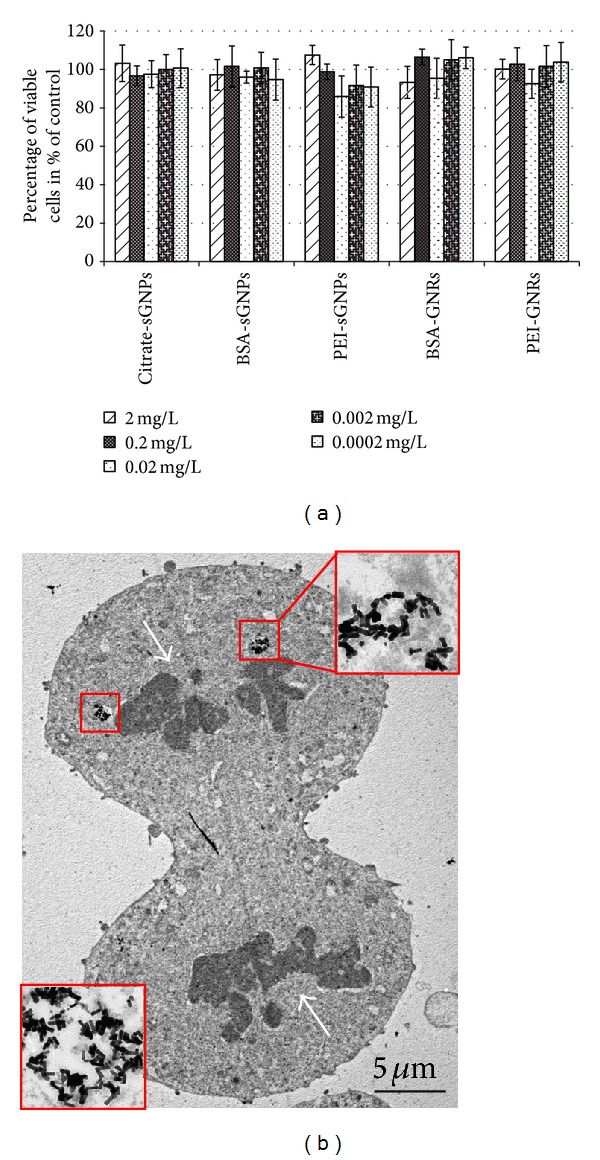
Cytotoxicity of GNPs determined by the MTT test. (a) Viability of HeLa (first two blocks) and BHK-21 cells after 48 h of incubation with different GNPs. Concentrations of GNPs are given under the horizontal axis. The vertical axis represents the percentage of viable cells. The viability of untreated cells was set as 100%. (b) Mitosis in the BHK-21 culture treated with PEI-GNRs. Arrows show chromosomes and boxes indicate PEI-GNRs in lysosomes. The inset shows an enlarged fragment of a lysosome filled with PEI-GNRs. TEM of ultrathin sections.

The high toxicity of CTAB-GNRs has been previously shown in many studies [25, 37]; therefore, we did not examine the cytotoxicity of these NPs. The results of the MTT tests demonstrated that the modification of both citrate-sGNPs and CTAB-GNRs by PEI did not increase the cytotoxicity in different cell lines as compared to GNPs modified with BSA- and citrate-sGNPs (Figure 4(a)). There were no differences in the cytotoxicity parameters of the studied GNPs between different cell lines, so we present data from the MTT assay for citrate- and BSA-sGNPs on HeLa cells and for PEI-sGNPs and PEI- and BSA-GNRs on BHK-21 cells. The electron microscopic study of BHK-21, B16, and HeLa cells after 24 h of incubation with GNPs showed the absence of cell damage. Moreover, cells treated with PEI-GNRs contained particles inside and continued to divide (Figure 4(b)). Taken together, our results show that PEI- and BSA-GNPs are nontoxic, independently of their size, shape, and surface charge.

### 3.4. TEM Study of GNPs Interaction with the Cells

The fine mechanisms of GNRs interactions with cells are poorly understood. The high toxicity of CTAB-GNRs makes examination of the mechanisms of their interaction with cells pointless. It is very important to understand the mechanism by which NPs penetrate cells; however, the vast majority of published studies of this topic have used fluorescence methods, which only show NPs penetration [5, 38]. In contrast, TEM allows the unambiguous identification of the structure of different types of endocytosis and various types of endosomes and lysosomes. We studied the interaction of nontoxic PEI-GNPs and BSA-GNPs with eukaryotic cells by TEM in order to better understand the role of surface coating and charge and GNP shape in the mechanisms of GNP-cell interactions.

**Figure 5:**
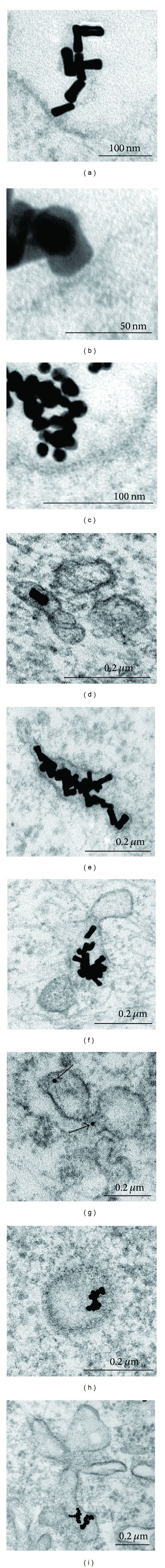
Adsorption and penetration of GNPs into the cells found after 30 min of incubation. (a), (b) The adsorption of PEI-GNRs on the surface of BHK-21 and B16 cells. Fibrillar material is visible between NPs and plasma membrane. (c) The adsorption of citrate-sGNPs on the plasma membrane of HeLa cells. (d) PEI-GNR in caveolae. (e), (f) PEI-GNRs in early endosomes. (g) PEI-sGNPs (shown by arrows) in caveolar clusters. (h) Citrate-sGNPs in a clathrin-coated vesicle. (i) Citrate-sGNPs in caveolar clusters. (d)–(g) BHK-21 cells; (h), (i) HeLa cells.

Firstly, we examined initial citrate-sGNPs. It was found that sGNPs adsorbed on the HeLa cell surface both individually and as loose agglomerates were separated from the membrane by a distance of 1-2 nm, with no signs of filaments being detected between the surface of cells and these GNPs (Figure 5(c)). Citrate-sGNPs entered HeLa cells by clathrin- and caveolin-mediated endocytosis (Figures 5(h) and 5(i)) and were detected in early endosomes after 30 min of incubation and in many lysosomes after 24 h of incubation.

The TEM study of ultrathin sections of BHK-21 and B16 cells after incubation with PEI-GNRs or PEI-sGNPs for 30 min showed high adsorption of branched chains or loose agglomerates of GNPs on the cells (Figure 5(a)), with no particles being found in the intercellular space. Fine filaments (1.5–2 nm in length) were seen in the contact areas between PEI-GNRs and the plasma membrane (Figure 5(b)), apparently reflecting the direct interaction of PEI-GNRs with filamentous molecules of the glycocalyx located on the outer surface of cell membranes. PEI-sGNPs were also separated from the plasma membrane at a distance of 1.5–2 nm; however, filaments were fuzzy in ultrathin sections due to the spherical shape of the particles.

**Figure 6:**
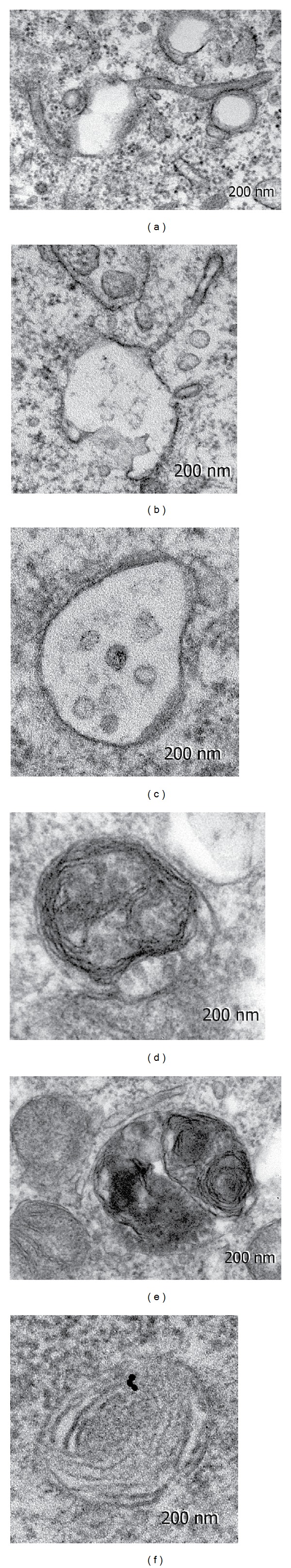
Representative images of (a), (b) early endosomes; (c) multivesicular body; (d), (e) lysosomes; and (f) melanosome. Distinct vesicular and tubular parts are seen in the photos of early endosomes. (a), (d), and (e) HeLa cells; (b), (c) BHK-21 cells; and (f) melanoma B16 cells. TEM of ultrathin sections.

The uptake of spherical and rod-shaped PEI-GNPs was evident after 30 min of incubation in ultrathin sections of both BHK-21 and B16 cells. PEI-GNRs were observed in caveolae and tubular lipid rafts, indicating caveolin-dependent and clathrin-caveolin-independent endocytosis, with no clathrin-dependent endocytosis being detected. The uptake of PEI-GNRs by BHK-21 cells was more active compared to that seen for B16 cells; GNRs were noted not only in caveolae (Figure 5(d)) but also in early (Figures 5(e) and 5(f)) and late endosomes. This was probably due to the high number of caveolae on the BHK-21 surface and the emergence of new caveolae; the number of caveolae per cell section increased by a factor of 1.5 in comparison with control cells. PEI-sGNPs were found in individual and clustered caveolae (Figure 5(g)), in occasional clathrin-coated pits and vesicles and in early endosomes of BHK-21 cells. Early endosomes are composed of vesicular and tubular parts (Figures 6(a) and 6(b)). B16 cells also internalised PEI-sGNPs, which were observed in tubular lipid rafts and early endosomes. Active accumulation of PEI-GNRs and PEI-sGNPs was observed in both BHK-21 and B16 cell lines after 3 h of incubation. Numerous particles and their aggregates were found in the late endosomes (multivesicular bodies). Multivesicular bodies (MVBs) were recognised by the presence of small intraluminal vesicles inside the cells (Figure 6(c)). MVBs accumulate and digest any internalised material and gradually mature into lysosomes, with the intraluminal vesicles remaining inside. Using TEM, we identified structures as lysosomes if they contained digested material inside the lumen (Figures 6(d) and 6(e)).

**Figure 7:**
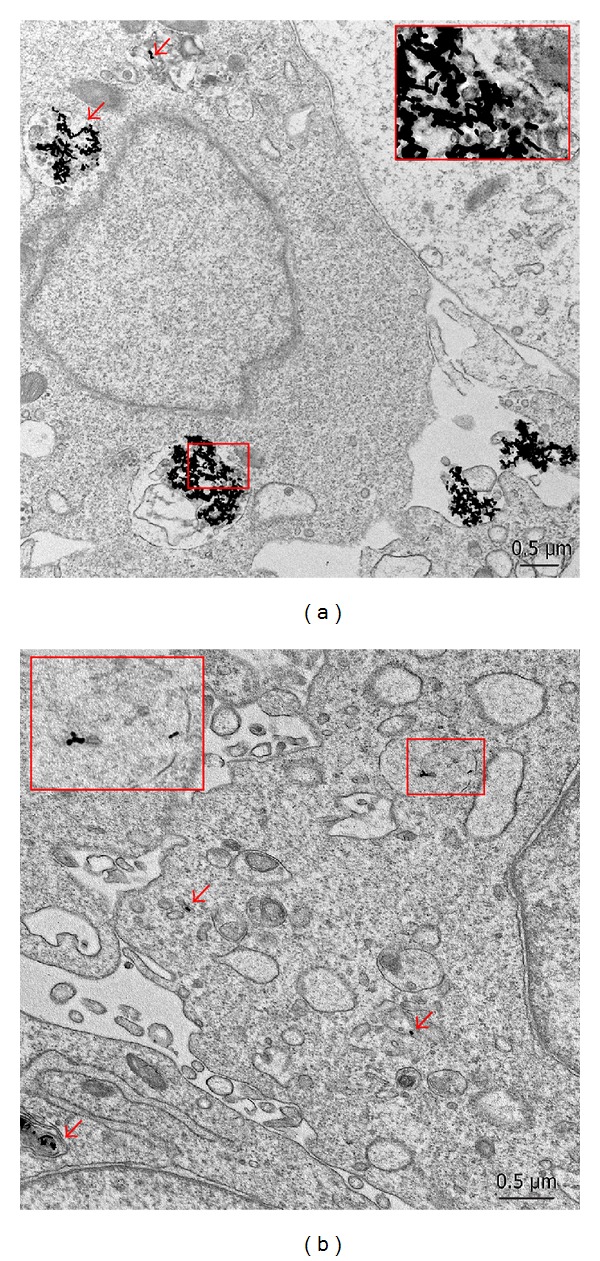
Differences in the accumulation of (a) PEI-GNRs and (b) BSA-GNRs in BHK-21 cells after 24 h of incubation. Insets show enlarged fragments of lysosomes containing GNRs. TEM of ultrathin sections.

The incubation of BHK-21 cells with spherical and rod-shaped PEI-GNPs for 24 h resulted in their accumulation in MVBs and lysosomes. The lysosomes and MVBs increased in size during incubation, and PEI-GNPs formed dense aggregates inside these structures (Figure 7(a)). The fusion of some PEI-GNRs-containing MVBs with the plasma membrane was noted in ultrathin sections. In B16 cells, PEI-GNPs were located in MVBs and occasionally in melanosomes (Figure 6(f)).

Surprisingly, GNRs and sGNPs coated with BSA were not found in ultrathin sections after 30 min of incubation with BHK-21 and HeLa cells, respectively, although the concentration of NPs in the media was identical to that in the case of PEI-GNPs and citrate-sGNPs. BSA-sGNPs were not found in ultrathin sections of cells after 3 h of incubation, while numerous citrate-sGNPs were observed in lysosomes. This observation indicates the long flotation of BSA-coated GNPs. Few BSA-GNRs bound to cell detritus were detected in B16 cells. Spherical and rod-shaped BSA-GNPs were not found at this time in the endocytic structures of any of the studied cell cultures, which provides further confirmation of the fact that positively charged NPs reach cells faster than negatively charged NPs [39]. After 24 h of incubation, BSA-sGNPs were observed in rare MVBs and lysosomes of HeLa cells, while citrate-sGNPs were stored mainly in numerous lysosomes.

**Figure 8:**
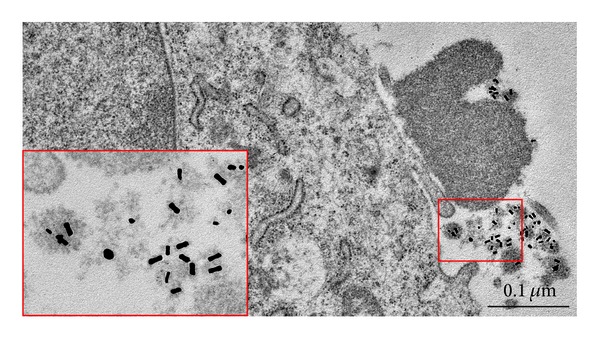
Representative images of BSA-GNRs associated with cell detritus (enlarged in the box) near the surface of a B16 cell after 3 h of incubation. TEM of ultrathin sections.

Binding with granular and filamentous components of cell detritus was a feature of BSA-GNRs, which were found after 3 h of incubation on the surface of the BHK-21 and B16 cells and in the intercellular space (Figure 8). The uptake of BSA-GNRs by both cell lines was incomparably lower than that of PEI-GNRs at the same time. Rare NR-containing clusters of caveolae and extremely rare NR-containing clathrin-coated vesicles were detected in BHK-21 cells. Phagocytosis of BSA-GNRs associated with components of cell detritus was observed in some B16 cells, which also contained rare early endosomes with a few NRs inside. Incubation of BSA-GNRs with BHK-21 and B16 cells for 24 h resulted in their accumulation in both cell lines; however, the total mass of BSA-GNRs was evidently smaller than that of PEI-GNRs in the same cells (Figure 7). B16 cells contained BSA-GNRs in lysosomes and melanosomes.

**Figure 9:**
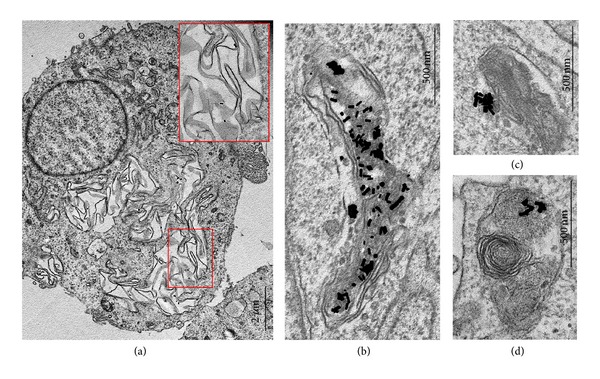
Representative images of large accumulations of membrane folds in BHK-21 cells after 3 h of incubation with BSA-GNRs. (a) GNRs can be seen on the membranes in the enlarged areas of the photo. (b)–(d) Lysosomes formed due to the compression of membrane accumulations and digestion of membranes, containing BSA-GNRs. TEM of ultrathin sections.

An interesting morphological phenomenon was observed in BHK-21 cells after 3 h of incubation with BSA-GNRs; that is, large accumulations of folded membranes related to individual GNRs were found in the cytoplasm (Figure 9(a)). These accumulations were observed in many cells and represented the internalisation of large areas of plasma membrane with adsorbed GNRs. Accumulations of membrane folds associated with BSA-GNRs decreased in size after 24 h of incubation, became electron dense, and acquired the structure of lysosomes (Figures 9(c) and 9(d)). The morphological parameters of the accumulations of membrane folds in the cytoplasm were identical to those in B16 cells treated with PEI-GNRs. It is possible to propose two mechanisms for the formation of these structures: macropinocytosis or endocytosis of circular dorsal ruffles. Both endocytosis pathways are responsible for the uptake of large plasma membrane areas [40, 41]. The mechanisms of both types of endocytosis are not yet understood, and the TEM descriptions of these two phenomena are poor. We observed the endocytosis of membrane folds in two types of cells, which interacted with different types of GNRs. While PEI-GNRs directly interacted with B16 cell membranes, BSA-GNRs were always bound to the components of cell detritus. We cannot determine whether the simultaneous direct interaction of numerous NPs with the cell surface induced the formation of membrane folds. It is also impossible to attribute the endocytosis of membrane folds to features of a particular cell line because the phenomenon was observed in both normal BHK-21 cells and B16 melanoma cells. We hypothesise that a mass of NPs simultaneously contacting the cell surface may be critical for the formation of membrane ruffles or protrusions. Indeed, after 30 min of incubation, B16 cells in ultrathin sections appeared heavily decorated by numerous PEI-GNRs adsorbed on their surface. The binding of BSA-GNRs to cell detritus may provide a mass necessary to induce the endocytosis of membrane folds observed in BHK-21 cells.

Thus, BSA-GNRs added to monolayers of BHK-21 and B16 cells showed prolonged flotation in the culture medium and first contacted components of cell detritus (Figure 8), which served as an intermediary agent and provided contact between GNRs and cells. It is likely that binding with cell detritus made BSA-GNRs heavy enough to overcome the repulsive force between BSA-GNRs and the cell surface. There is an opinion that as soon as any NP is immersed in cell culture medium, it is immediately covered by peptides and other biomolecules [28, 31, 38]. The DLS data show that both BSA- and PEI-GNRs acquired a negative charge in DMEM containing FBS (Figure 2); however, PEI-GNRs showed faster and more efficient adsorption and penetration into cells, in contrast with BSA-GNRs (Figure 7). We assumed that the electric charge caused by the surface modification (with BSA or PEI) might be the main factor responsible for this, which determines the interaction of NPs with the cell surface. This conclusion supports the suggestion of Hühn and coauthors that differences in NP interactions with cells depend on the surface charge, rather than on the presence of a corona [39].

## 4. Conclusions 

Recent studies have shown that the behaviour of nanoparticles in biological media is more complicated than what was previously thought and conceptually different from known colloidal phenomena. The surface charge is very important for their uptake by cells, and positively charged NPs have a high affinity for negatively charged cell membranes [42–44], independently of the presence of serum in the cell culture medium [35]. We examined the physicochemical parameters of PEI- or BSA-modified spherical and rod-shaped GNPs and their behaviour in different liquids used for cell propagation. Our study showed that the adsorption and uptake of GNPs by different cells depends on the electric charge of GNPs, which was determined by a modifying substance (PEI or BSA). We may therefore conclude that the principal role belongs to the charge of initial NPs. Thus, the positive charge of PEI-GNPs changed to negative in DMEM and conditioned DMEM; however, these nanoparticles efficiently penetrated into cells. It is worth noting that lysosomes were the final destination for all of the studied GNPs, which remained enclosed inside these structures and did not enter the cytoplasm at least within 24 h of incubation.

The ability to penetrate cells without causing damage to the cell structure is very important for the application of NPs in nanobiotechnology. The modification of NPs with PEI allowed us to overcome the problem of the high toxicity of CTAB-GNRs. These novel PEI-GNRs are nontoxic and stable in cell culture media and can efficiently penetrate into normal and malignant cells through caveolin-dependent and lipid raft-mediated endocytosis.

## Figures and Tables

**Table 1:** Electron microscopic characterisation of the dispersity of GNPs in different liquids.

NPs/medium	Aqueous solution	Phosphate buffer	10% FBS in water	DMEM with 10% FBS	Conditioned DMEM
Citrate-sGNPs	Individual NPs	n.d.∗∗∗	n.d.	Agglomerates∗∗	Aggregates∗
BSA-sGNPs	Individual NPs	Agglomerates	Individual NPs	Individual NPs	Individual NPs
PEI-sGNPs	Individual NPs	Aggregates	Individual NPs	Individual NPs	Individual NPs
CTAB-GNRs	Individual NPs	n.d.	n.d.	Individual NPs	Individual NPs
BSA-GNRs	Aggregates	Aggregates	Agglomerates	Individual NPs	Agglomerates
PEI-GNRs	Agglomerates	Aggregates	Individual NPs + agglomerates	Individual NPs + agglomerates	Individual NPs

*Aggregates are clusters of NPs tightly adjacent to each other with invisible gaps between them.

∗∗Agglomerates are loose clusters of NPs comprising more than 10 units with gaps of less than 2 nm between them, which can be seen by TEM.

∗∗∗n.d.: not determined.
